# Epidemiological Study of the Relationship Between Antimicrobial Resistance Genes and Biofilm-Forming Capacity in Pathogens Causing Chronic Wound Infections

**DOI:** 10.3390/microorganisms14051117

**Published:** 2026-05-14

**Authors:** Silvia Ioana Musuroi, Adela Voinescu, Corina Musuroi, Delia Muntean, Florin George Horhat, Luminita Mirela Baditoiu, Oana Izmendi, Andrei Cosnita, Valentin Ordodi, Zorin Crainiceanu, Edward Seclaman, Monica Licker

**Affiliations:** 1 Doctoral School, “Victor Babes” University of Medicine and Pharmacy, 300041 Timisoara, Romania; silvia.musuroi@umft.ro (S.I.M.); adela.voinescu@umft.ro (A.V.); oana.izmendi@umft.ro (O.I.); 2 Multidisciplinary Research Center on Antimicrobial Resistance (MULTI-REZ), “Victor Babes” University of Medicine and Pharmacy, 300041 Timisoara, Romania; horhat.florin@umft.ro (F.G.H.); baditoiu.luminita@umft.ro (L.M.B.); crainiceanu.zorin@umft.ro (Z.C.); licker.monica@umft.ro (M.L.); 3 Discipline of Clinical Skills, Department I Nursing, “Victor Babes” University of Medicine and Pharmacy, 300041 Timisoara, Romania; 4 Microbiology Department, “Victor Babes” University of Medicine and Pharmacy, 300041 Timisoara, Romania; 5 Microbiology Laboratory, “Pius Branzeu” County Clinical Emergency Hospital, 300723 Timisoara, Romania; 6 Clinical Laboratory, “Louis Turcanu” Emergency Hospital for Children, 300011 Timisoara, Romania; 7 Epidemiology Department, “Victor Babes” University of Medicine and Pharmacy, 300041 Timisoara, Romania; 8 Surgery & Ophthalmology Department, “Victor Babes” University of Medicine and Pharmacy, 300041 Timisoara, Romania; cosnita.dan@umft.ro; 9 Center of Immuno-Physiology and Biotechnologies, Department of Functional Sciences, “Victor Babes” University of Medicine and Pharmacy, 300041 Timisoara, Romania; valentin.ordodi@umft.ro; 10 Research Center for Gene and Cellular Therapies in the Treatment of Cancer—OncoGen, “Pius Brinzeu” County Clinical Emergency Hospital, 300011 Timisoara, Romania; 11 Plastic Surgery Department, “Victor Babes” University of Medicine and Pharmacy, 300041 Timisoara, Romania; 12 Biochemistry Department, “Victor Babes” University of Medicine and Pharmacy, 300041 Timisoara, Romania; eseclaman@umft.ro

**Keywords:** chronic wounds, antibiotic resistance genes, biofilm-forming isolates, biofilm resistance to antibiotics

## Abstract

Chronic wounds represent a major complication of underlying conditions such as diabetes mellitus, arterial ischemia, surgical wound and burns. This study aimed at the phenotypic and molecular characterization of antimicrobial resistance for a selection of bacterial isolates, originating from wounds harvested from patients hospitalized in the Vascular Surgery and Plastic Surgery wards. The microbiological diagnosis of wound infections was established according to the laboratory’s working protocol. PCR screening of antibiotic resistance genes was performed using a real-time PCR, while the microtiter plate assay was used to determine the biofilm-forming capacity. Testing of biofilm susceptibility to meropenem and amikacin was performed on Calgary biofilm device. Of the 88 bacterial isolates studied, 78.40% were Gram-negative bacilli (GNB)—*Klebsiella pneumoniae* (K.P), *Pseudomonas aeruginosa* (P.A), *Proteus mirabilis* (P.M), *Acinetobacter baumannii* (A.B), while the remaining 21.60% were Gram-positive cocci (GPC)—*Staphylococcus aureus* (S.A). All A.B isolates and 92.59% of K.P were carriers of β-lactamase- and carbapenemase-encoding genes, while 57.89% of *S. aureus* isolates were carriers of *mecA* (methicillin-resistant). Strong biofilm-forming isolates (B+++) were more frequent in P.A than in K.P (*p* = 0.002) and P.M (*p* = 0.02), with a frequency comparable to that of A.B strains (*p* = 0.212). When analyzing the biofilm reaction to meropenem, a significantly lower susceptibility was detected in the biofilm for K.P isolates, compared to the planktonic ones. Most GNB have been extensively multidrug-resistant, particularly K.P and A.B. Isolates from chronic wounds are major biofilm-formers. A strong and statistically significant association has been identified in the case of K.P and P.M between the presence of resistance genes and the biofilm-forming capacity. These findings highlight the need for a customized therapeutic approach for each chronic wound, considering the mechanisms underlying treatment resistance. These include bacterial virulence factors and the wound microenvironment colonized by the biofilm and the relative contribution of each to the overall resistance profile.

## 1. Introduction

Chronic wounds are a group of lesions, for which the healing process deviates from the normal, progressive, and time-limited stages of repair. As a result, they persist in the inflammatory stage of evolution with delayed healing for a long time. A wound is considered chronic when the healing process exceeds 4–6 weeks [1].

The importance of chronic wounds is given by their high prevalence—up to 86.70/10.000 inhabitants according to certain studies (2018), by the long-term impact on the life quality of patients, and by the high cost that burdens health systems [2].

Most chronic wounds are divided into three categories: diabetic foot ulcers, venous ulcers, and pressure ulcers. In their case, certain processes that could underlie the phenomena slowing down healing, such as local tissue hypoxia, repetitive ischemia-reperfusion injuries, altered cellular and systemic stress response, and bacterial colonization have been identified [3]. Another category is that of injuries from surgical and burn wounds, in which case the healing process is determined by additional causal factors that are defined by the surgical disease, as well as by the dimensions of the burned surfaces.

Several clinical studies related to the physiopathological and microbiological phenomena of wound healing indicate that the main pathogens involved in the healing delay of chronic wounds are the Gram-positive cocci (GPC) *Staphylococcus aureus*, beta-hemolytic streptococci, and the Gram-negative bacilli (GNB) *Pseudomonas aeruginosa*, *Acinetobacter baumannii*, and *Enterobacterales* species (*Proteus mirabilis*, *Klebsiella pneumoniae*, *Escherichia coli*), as well as anaerobic pathogens insufficiently identified and investigated in these pathologies [4,5,6]. It has also been demonstrated that the microbiota of chronic non-healing wounds of patients treated with antibiotics are significantly different from that of untreated patients, which could point to the conclusion that antimicrobial therapy leads to the alteration of the community structure through the numerical reduction in some species with the selection of others [3].

Repeated hospitalization for chronic wound disease is marked by the risk of infection with hospital-acquired strains, mainly multidrug-resistant GNB (*Acinetobacter baumannii*, *Klebsiella pneumoniae*) [7,8]. The repair and healing stages of chronic wounds can also be compromised by the pathogenic mechanisms of these diseases.

Regardless of their etiology, the population of chronic non-healing wounds with biofilm-forming strains is an important aggravating factor. The bacterial biofilm in the wound induces tissue hypoxia through microbial metabolic activities and promotes a hyperinflammatory response detrimental to the host [9,10]. Recent studies have indicated that once the biofilm has been established, it fosters wound chronicity through resistance to antimicrobial treatment and the action of disinfectants, as well as through resistance to the host immune defense [11]. It is becoming more widely accepted that biofilms are detected in most chronic non-healing wounds, with some studies indicating high frequencies of over 78% [11]. At the same time, it is increasingly stressed that microbiological investigations of chronic wounds have limited diagnostic performance because they provide partial identification of infectious agents over a period exceeding 24 h. There are studies which have highlighted population diversity patterns in chronic wounds and have introduced the concept of functionally equivalent pathogroups (FEPs). At the same time, it has been shown that traditional culture methods can be highly biased as a diagnostic tool, in which case high-resolution molecular diagnostic methods such as bacterial tag-encoded FLX amplicon pyrosequencing (bTEFAP) are suggested [6,12].

This study aimed to investigate the antimicrobial resistance genes and the biofilm-forming capacity of the bacterial isolates from chronic wounds of patients in our hospital wards. The species were selected based on the results of previous studies [7,13]. It also aimed to highlight the need for a customized therapeutic approach to chronic wounds, considering the mechanisms underlying treatment resistance. These include bacterial factors (such as resistance gene carriage and biofilm-forming capacity), as well as factors related to the wound microenvironment (colonized by the biofilm), and the relative contribution of each to the overall resistance profile.

## 2. Materials and Methods

This research is a cross-sectional analytical study that was carried out in the Vascular Surgery and Plastic Surgery wards of the “Pius Brinzeu” County Clinical Emergency Hospital Timișoara (SCJUPBT) and the Research Center of the “Victor Babeș” University of Medicine and Pharmacy Timișoara (UMFT). The medical unit is a tertiary teaching hospital, affiliated to the university, equipped with 1174 beds, which provides medical care for the western region of Romania. Sample collection was carried out between 1 November 2023 and 30 May 2024 and laboratory tests were completed by the end of 2024.

The study aimed to molecularly identify selected antibiotic resistance genes, describe antimicrobial resistance phenotypes based on the antibiogram results, and phenotypically assess the biofilm-forming capacity of the strains. It also aimed to investigate the associations between resistance gene carriage and the biofilm-forming ability, followed by the evaluation of biofilm susceptibility to selected antibiotics.

The selected strains were *Klebsiella pneumoniae*, *Acinetobacter baumannii*, *Pseudomonas aeruginosa*, *Proteus mirabilis* (GNB), and *Staphylococcus aureus* (GPC), pathogenic species with high frequencies in the wards mentioned.

The selected samples were part of the working samples of patients admitted to the hospital wards, and the antibiotic sensitivity study was conducted as part of the customary microbiological diagnosis. The selection of strains for this study was carried out in real time, during routine diagnostic procedures, according to the inclusion criteria.

Samples from chronic wounds were collected after cleaning the wound with sterile saline solution and removing necrotic tissue and superficial secretions. Samples were collected from the deep, viable area of the wound, taking care to avoid contamination with skin flora. Sterile ESwab collection kits (Copan, Brescia, Italy) containing 1 mL of Amies liquid transport medium were used. The sterile swab was applied to the granulation tissue and rotated. Each sample collected that way was transported to the laboratory and subsequently processed according to standard microbiological diagnostic procedures.

The minimum inhibitory concentration (MIC) for planktonic bacteria was determined using standardized antimicrobial susceptibility tests such as microdilution tests on the automated VITEK^®^ 2 Compact (BioMerieux, Marcy l’Étoile, France) system. We used a standardized inoculum of bacteria (10^5^ CFU/mL) and we followed the EUCAST 2024 guidelines [14] and manufacturer’s instructions in terms of incubation temperature, time, medium. Also, E-test strips (Thermo Fisher Scientific, Waltham, MA, USA) were used when Vitek results were inconclusive (gradient diffusion method).

The results obtained from routine microbiological testing were reported for the purpose of initiating antibiotic therapy, and among the isolated strains, those that met the study inclusion criteria were selected for the research activities described.

The selected strains were cryopreserved at −78 °C in a brain heart infusion broth (BHI-Thermo Fisher Scientific, Oxoid Deutschland GmbH, Wesel, Germany), in a UNYCRO DEEP FREEZER (−85 °C, UniEquipP, Munich, Germany). The research was carried out once the complete sample set was collected.

The specific criteria for inclusion in the study were of a clinical and microbiological nature: (i) the wound considered for collection must be older than 4 weeks, (ii) only pathogens isolated from the first sample collected during the current hospitalization would be considered, and (iii) only pathogens of interest would be considered (*Klebsiella pneumoniae*, *Acinetobacter baumannii*, *Pseudomonas aeruginosa*, *Proteus mirabilis*, *Staphylococcus aureus*).

Written informed consent was obtained from all patients (or their legal representatives, when applicable) for wound sampling and the use of their clinical and paraclinical data for research purposes. All data were anonymized prior to analysis to ensure patient confidentiality.

### 2.1. Analysis of Resistance Phenotypes

Isolate identification, antibiogram, and determination of the minimum inhibitory concentration (MIC) were carried out according to the working protocol of the Microbiology Department of the Clinical Laboratory of Medical Analysis, using E-tests, the VITEK^®^ 2 Compact (BioMerieux) and Matrix-assisted Laser Desorption/Ionization Time-of-Flight Mass Spectrometry (MALDI Biotyper, Bruker Daltonics, Bremen, Germany) systems. The interpretation of the antibiograms was carried out according to the EUCAST 2024 guidelines [14].

The study strains were described according to their antibiotic resistance phenotypes, as follows: (1) Methicillin-resistant *S. aureus* (MRSA): *S. aureus* resistant to oxacillin/cefoxitin according to EUCAST guidelines [14]. (2) Extended-spectrum β-lactamase (ESBL)-producing Gram-negative bacilli (GNB): resistant to third/fourth-generation cephalosporins and monobactams, confirmed by synergy test. (3) Carbapenem-resistant (CR) GNB: defined by phenotypic resistance to at least one carbapenem (imipenem, meropenem, or ertapenem), according to the EUCAST guidelines [14].

The epidemiological phenotypes, MDR and XDR, were defined according to the criteria of Magiorakos et al. [15].

### 2.2. PCR Screening of Antibiotic Resistance Genes

Gram-negative isolates were tested for the presence of beta-lactam, carbapenem, aminoglycoside resistance genes, while Gram-positive isolates were tested for beta-lactam resistance, methicillin resistance (MR), macrolide, fluoroquinolone, and aminoglycoside resistance. The primers used in this study are illustrated in Table 1.

Microbial genomic DNA was extracted from overnight bacterial cultures using PureLinkTMMicrobiome Purification Kit (Thermo Fisher Scientific), according to the manufacturer’s instructions (User Guide, Publication No. MAN0014266).

Detection of antimicrobial resistance genes was performed using a real-time PCR assay with TaqMan hydrolysis probes on an Azure Cielo Real-Time PCR system (Azure Biosystems, Inc., 6747 Sierra Court, Suite A–B, Dublin, CA, USA). The assay targeted a selected panel of clinically relevant resistance genes and was designed exclusively for qualitative detection (presence/absence).

Lyophilized primers were resuspended in ultrapure water to obtain stock solutions of 100 μM, and working solutions were prepared at 20 μM and stored at −20 °C until use. Each reaction was performed in a final volume of 20 μL, containing 10 μL of 2 × PCR master mix, 0.5 μM of each primer, 0.25 μM TaqMan probe, 3 μL of purified DNA template and nuclease-free water to complete the reaction volume. For each run, the reaction mix was prepared for all samples plus two additional reactions to compensate for pipetting loss. A volume of 17 μL of reaction mix was dispensed into each well, followed by 3 μL of DNA template.

Amplification was performed under the following conditions: initial denaturation at 95 °C for 2 min, followed by 40 cycles of denaturation at 95 °C for 15 s and annealing/extension at 65 °C for 20 s, with fluorescence acquisition. Fluorescence signals were recorded in real time using the appropriate detection channel (e.g., FAM).

Each real-time PCR run included positive controls, consisting of DNA from reference strains known to harbor the target resistance genes and negative controls (no-template controls) to monitor contamination. All samples were analyzed in duplicate to ensure reproducibility.

Results were interpreted qualitatively based on amplification curves and cycle threshold (Ct) values. Samples were considered positive when a characteristic amplification curve was observed with a Ct value below the predefined threshold. Samples with no amplification or Ct values above the threshold were considered negative.

No standard curve was generated, and no quantitative analysis of gene expression or copy number was performed. Ct values were used only for qualitative interpretation and not for comparative or quantitative analysis.

### 2.3. Determination of the Biofilm-Forming Capacity

Determination of the biofilm-forming capacity was performed using the microtiter plate technique [24]. Sterile, flat-bottom, polystyrene plates with 96 wells were used.

The strains isolated from the agar plates were inoculated into 2 mL BHI broth supplemented with 1% glucose and incubated at 37 °C for 24 h. In the next stage, a 1:100 dilution was made with a fresh culture medium, after which 200 µL of the diluted culture was inoculated into each well of the plates. The plates were incubated for 24 h at 37 °C.

The content of the wells was subsequently removed, the plates were washed three times with 0.2 mL phosphate-buffered saline (PBS, pH 7.2). The plates were incubated for 1 h at 60 °C and then stained with 1% crystal violet. The excess dye was removed by rinsing three times with deionized water, after which decolorization with 30% acetic acid was performed. The absorbance (optical density, OD) of the stained adherent biofilm was measured with an automated micro-ELISA plate reader, at a wavelength of 570 nm. For QC, we used *Pseudomonas aeruginosa* ATCC 27853, and *Staphylococcus aureus* ATCC 29213 [25]. Each strain was tested in 3 wells. The uninoculated wells, which contained only broth served as the negative control. The mean OD values were calculated for all tested strains and negative controls. Biofilm production was interpreted in accordance with the criteria suggested by Stepanović et al. [24] and other studies using the same classification [26].

Isolates from wells with OD values higher than those of the negative control were considered biofilm producers. Biofilm production was defined according to the ODc classification, where ODc represents the mean optical density of the negative control plus three standard deviations. Isolates were classified as non-biofilm producer (OD ≤ ODc), weak biofilm producer (ODc < OD ≤ 2 × ODc), moderate biofilm producer (2 × ODc < OD ≤ 4 × ODc) and strong biofilm producer (OD > 4 × ODc). To indicate the results, we used (+++) for strong biofilm-forming strains, (++) for moderate biofilm-forming strains, (+) for weak biofilm-forming strains, and (0) for strains that did not form biofilm [24].

### 2.4. Biofilm Susceptibility Testing to Antibiotics

#### 2.4.1. Preparation of Antibiotic Stock Solutions

We used meropenem (Meropenem, USP M002-50 mg, Toku-E, Centennial, CO, USA) and amikacin (Amikacin A002-1G, Toku-E, Centennial, CO, USA) for biofilm strain testing. A stock solution was prepared with a concentration of 10 mg/mL and stored at −20 °C until it was ready for use. Serial dilutions of 0.125–128 µg/mL were prepared.

#### 2.4.2. Biofilm Susceptibility Testing to Meropenem and Amikacin

This was performed using the Calgary biofilm device (MBEC Assay^®^ Biofilm Inoculator with 96 Well Base, 19113, Innovotech, Edmonton, AB, Canada) for biofilm testing in accordance with a revised protocol described by Pandelis et al. [27]. Therefore, 100 μL of a 1:100 dilution of an overnight culture was added to 100 μL of BHI broth supplemented with 1% glucose in each well of a 96-well Calgary biofilm device. The lid was placed so that the pegs could be immersed in the wells. The plates were incubated at 37 °C for 48 h with gentle shaking to allow biofilm formation on the surface of the pegs.

The peg lids were then subjected to 2 consecutive washes to remove planktonic organisms by consecutively submersing the pegs in two 96-well plates containing phosphate-buffered saline (PBS). After the second wash, the lids were placed in another 96-well plate containing serial dilutions of both meropenem and amikacin, in 3 wells each, and antibiotic-free wells for positive control. The plates were incubated at 37 °C for 48 h with shaking.

The post-exposure wash was performed 2 times with PBS. The peg lids were then placed in a recovery medium plate, and the biofilm was detached by vortexing to release the biofilm into the well medium. The peg lids were discarded and replaced with sterile standard lids and the reading was performed (crystal violet staining and OD measurement). Biofilm formation was confirmed by bacterial growth observed in the antibiotic-free positive control wells.

In this study, we have determined the BMIC_50_ value defined as the lowest antimicrobial concentration that inhibits 50% of the biofilm growth compared to the growth observed in positive control wells, also used in other studies [27]. This endpoint was selected because biofilm responses to antimicrobial exposure are heterogeneous and often gradual rather than binary, making complete inhibition thresholds less robust and more difficult to define reproducibly [28]. The use of BMIC_50_ reduces the influence of outliers and biological variability of the strains, thereby providing a more robust and comparable estimation of the antibiotic activity against biofilms. Consequently, BMIC_50_ was considered more suitable for comparative assessment of antibiofilm activity in the present study.

### 2.5. Statistical Method

The database was analyzed via the IBM SPSS Statistics 20 program (SPSS Inc., Chicago, IL, USA). Nominal variables were expressed as values and percentages and comparisons were performed using the Chi-squared test (Fisher’s exact test). To assess the strength of the association between the presence of resistance genes and the biofilm-forming capacity, the Cramer’s V test was applied. The threshold for statistical significance was set at ≤0.05 and all tests were two-tailed.

The normality of the distribution of differences in MIC values between planktonic and biofilm strains was assessed using a Shapiro–Wilk test. Since the distribution deviated significantly from normality (*p* < 0.05), the differences between BMIC_50_ (for biofilm form of strains) and MIC values (for planktonic form), obtained when testing the susceptibility to the two antibiotics (MEM, AK), were analyzed using the Wilcoxon Signed-Rank test for paired samples. Effect size for Wilcoxon tests was calculated with the formula r = z/√N, where “z” is the statistical value of the test and “N” is the number of pairs.

The threshold for statistical significance was set at ≤0.05 and all tests were two-tailed.

## 3. Results

### 3.1. Chronic Wound Characteristics and Bacterial Profile

Based on the sample and species selection criteria, the study sample consisted of 88 bacterial strains, of which 69 (78.40%) were Gram-negative strains such as *K. pneumoniae*, *P. aeruginosa*, *P. mirabilis*, and *A. baumannii*, while 19 (21.60%) were Gram-positive strains represented by *S. aureus*. The distribution is presented in Table 2.

Among Gram-negative isolates, *K. pneumoniae* and *P. aeruginosa* were identified at comparable frequencies and were significantly more prevalent than *P. mirabilis* and *A. baumannii*.

*S. aureus* isolates were also significantly more frequent than *A. baumannii* isolates, while exhibiting frequencies comparable to those of *K. pneumoniae* and *P. aeruginosa*.

**Figure 1:**
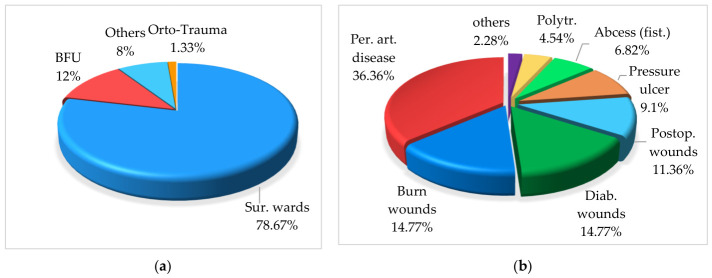
Distribution of wound samples (%): (**a**) by hospital ward, (**b**) by etiology. Legend: (**a**) Sur. wards: surgical wards, BFU: Burn Functional Unit, Orto-Trauma: Orthopedics–Traumatology; (**b**) Per. art. disease: peripheral arterial disease, Diab. wounds: diabetic wounds, Postop. wounds: postoperative wounds, Abscess (fist.): chronic abscess with fistula, Polytr.: polytrauma wounds.

The majority of wound samples originated from the Surgery ward (78.66%), followed by the Burn Unit (12%) while a small proportion was collected from the Orthopedics–Traumatology unit (1.33%) (Figure 1a). Other departments (8%) are represented by medical wards where these patients were surgically cared for (Neurology, Diabetes and Nutrition).

Wounds were most commonly associated with peripheral arterial disease (36.36%), followed by diabetic and burn wounds (14.77% each) and postoperative wounds (11.36%), while other etiologies were less frequent (Figure 1b).

**Figure 2:**
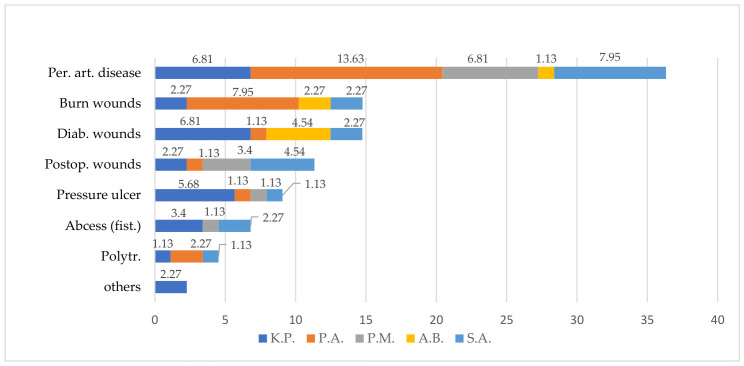
Distribution of microorganisms across wound samples (%). Legend: Per. art. disease: peripheral arterial disease, Diab. wounds: diabetic wounds, Postop. wounds: postoperative wounds, Abcess (fist.): Chronic abscess with fistula, Polytr.: politrauma wounds, K.P: *K. pneumoniae*, P.A: *P. aeruginosa*, P.M: *P. mirabilis*, A.B: *A. baumannii*, S.A: *S. aureus*.

The distribution of bacterial isolates varied according to wound etiology (Figure 2). Gram-negative pathogens, particularly *P. aeruginosa* and *K. pneumoniae*, were predominantly associated with wounds related to peripheral arterial disease, where the highest isolation frequencies were observed. *P. aeruginosa* was also frequently identified in burn wounds, while *K. pneumoniae* showed a more uniform distribution across several wound types, including diabetic and pressure ulcers.

*A. baumannii* was mainly associated with diabetic and burn wounds, whereas *S. aureus* was more frequently isolated from postoperative wounds and wounds associated with peripheral arterial disease. Overall, peripheral arterial disease-related wounds exhibited the greatest microbial diversity and burden.

### 3.2. Resistance Genes

The antimicrobial resistance genes identified in Gram-negative species are presented in Table 3.

*K. pneumoniae* strains were significant carriers of *bla*genes (92.59%), encoding both ESBLs and carbapenemases. The frequency of β-lactamase-encoding genes (TEM, SHV, CTX-M) was statistically comparable between strains; moreover, 66.66% (*n* = 18) of the *K. pneumoniae* strains carried the entire group of ESBLs: TEM, SHV, CTX-M, (class-A beta-lactamases, Ambler classification) [29]. The most frequent carbapenemases-encoding genes (*bla* genes–carba) were NDM (Ambler class-B) and OXA48 (Ambler class-D), with statistically comparable incidence (*p* = 0.09, OR = 2.96, 95% CI: 0.85–10.65). Their simultaneous presence in the same strain was identified in 40.74% (*n* = 11) of the isolates.

The association of *bla*NDM with at least one *bla*OXA gene was recorded in 48.14% (*n* = 13) of the *K. pneumoniae* strains. These *bla*NDM–*bla*OXA-carrying strains also associated at least one of the three class-A genes: TEM, SHV or CTX-M. *K. pneumoniae* strains did not carry *bla*VIM nor AG-resistance gene *aac(6′)-Ib*.

Regarding *P. aeruginosa* strains, 50% were carriers of resistance genes. All types of β-lactamase-encoding genes were identified, with *bla*TEM being the most frequently detected; however, the difference was not statistically significant.

The identified carbapenemase *bla* genes belonged exclusively to the OXA group, with two isolates harboring the OXA-23/OXA-24 association in the absence of other genes. All isolates carrying an OXA-type gene also harbored at least one additional β-lactamase gene (20.83%, *n* = 5). Two strains carrying the AG-resistance gene *aac(6′)-Ib* were identified.

*P. mirabilis* strains were frequently found to carry the *bla*TEM gene (72.72%, *n* = 8), with two of these isolates also harboring the CTX-M gene. Three isolates were carriers of *bla*genes—carba, all in association with *bla*TEM; the AG resistance gene was identified in two strains. No isolates carrying *bla*OXA-24 or *bla*VIM were detected. The strains with multiple carriage (*n* = 2) presented the associations *bla(*TEM/NDM/OXA48)/*aac(6′)–Ib* and *bla(*TEM/CTX-M/OXA23)/*aac(6′)–Ib*.

*A. baumannii* strains were all carriers of resistance genes, primarily *bla*TEM and *bla*OXA-23. Two strains were carriers of multiple genes. The first associated SHV/CTX-M/NDM, while the second carried TEM/OXA23/OXA24/OXA48.

These findings indicated that *K. pneumoniae*, *A. baumannii*, and *P. mirabilis* were the species with the highest carriage rates of β-lactamase genes, with no statistically significant differences observed between them. The species with the lowest carriage was *P. aeruginosa*. Concerning the carbapenem-resistance genes, they were identified for *K. pneumoniae* and *A. baumannii* strains, significantly more numerous compared to *P. aeruginosa* and *P. mirabilis* (K.P./P.A.: *p* = 0.002, OR = 7.00, 95% CI: 1.74–29.45), A.B/P.A., *p* = 0.028, OR = 12.00, 95% CI: 1.07–585.42; K.P./P.M: *p* = 0.008, OR = 9.33, 95% CI: 1.51–67.56, A.B./P.M, *p* = 0.05, OR = 16.00, 95% CI: 1.00–824.97).

Of all isolates included in the study, 17 did not carry any of the investigated antibiotic resistance genes: 2 *K. pneumoniae*, 12 *P. aeruginosa*, or 3 *P. mirabilis*. All *A. baumannii* isolates carried at least one resistance gene.

Concerning *S. aureus* strains, 57.89% (*n* = 11) indicated at least one resistance gene. The *MecA* strains were detected in most carrier strains, whereas associations with other genes was observed only sporadically (*mecA*—*aac(6′)aph(2″)*, 10.52%, *n* = 2). The resistance genes identified in *S. aureus* isolates are shown in Table 4.

It was noticed that the number of *S. aureus* isolates carrying resistance genes was significantly lower than that observed for *K. pneumoniae* isolates (*p* = 0.009, OR = 0.11, 95% CI: 0.01–0.70). However, no statistically significant differences were observed compared to *P. aeruginosa, P.mirabilis* and *A. baumannii* isolates. Nevertheless, the carriage rate among *S. aureus* isolates remained notable, with 72.72% carrying a single resistance gene.

### 3.3. Resistance Phenotypes

The antimicrobial resistance phenotypes of the Gram-negative strains are shown in Table 5.

All ESBL-*K. pneumoniae* strains (77.77%, *n* = 21) were non-carbapenemase *bla*genes carriers. All were equipped with *bla*SHV, with 85.71% (*n* = 18) of them showing the association TEM/SHV/CTX-M. Among the strains exhibiting a carbapenem-resistant phenotype (74.07%, *n* = 20), 95% (*n* = 19) carried at least one carbapenem resistance gene, with *bla*NDM detected in 90% (*n* = 18), and *bla*OXA48 in 60% (*n* = 12) of the isolates. Only one carbapenem-resistant strain was devoid of *bla* genes-carba; however, it carried the SHV and CTX-M genes. The aminoglycoside-resistance (AG-R) phenotype was detected in approximately 50% of the strains, although no strains carrying the *aac(6′)-Ib* resistance gene were identified.

*Pseudomonas aeruginosa* strains (*n* = 24) exhibited a more restricted antimicrobial resistance phenotype compared to *K. pneumoniae* isolates (Table 5). The two XDR isolates carried TEM in association with OXA23/OXA24, one of them also harboring SHV and CTX-M genes. Regarding the MDR phenotype (41.99%, *n* = 10), no resistance genes among those investigated were identified in half of the strains (*n* = 5). Among the carbapenem-resistant isolates, carbapenemase genes were detected in all but one strain, which exhibit carbapenem resistance only phenotypically. Furthermore, aminoglycoside resistance in three *P. aeruginosa* strains was detected only phenotypically, in the absence of the investigated resistance gene. Notably, the two isolates carrying the *aac(6′)-Ib* gene remained phenotypically susceptible to aminoglycosides (GN, AK or TOB).

The majority of *P. mirabilis* strains exhibited an MDR phenotype. The aminoglycoside resistance phenotype was detected in three isolates, although only one carried the *aac(6′)-Ib* gene. Conversely, one gene-carrying isolate did not exhibit the AG-R phenotype.

The majority of *A. baumannii* strains belonged to the carbapenem-resistant XDR type, associating the AG-R phenotype. Only one isolate carried the *aac(6′)-Ib* gene.

Among the *S. aureus* isolates, 52.63% (*n* = 10) were methicillin-resistant, while 68.42% (*n* = 13) exhibited a multidrug-resistant (MDR) phenotype.

### 3.4. Biofilm Formation

**Figure 3:**
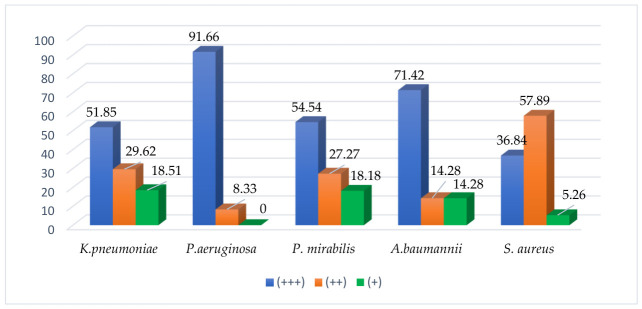
Biofilm-forming capacity of strains (%).

The biofilm-forming behavior of Gram-negative strains is shown in Figure 3. All bacteria examined were biofilm-formers, with different intensity and proportions depending on the species. It was observed that most strains are strong/medium biofilm-forming (at least 80% isolates of each species).

Strong biofilm-forming strains (B+++) were more frequent in the case of *P. aeruginosa* isolates compared to *K. pneumoniae* (*p* = 0.002, OR = 0.09, 95% CI: 0.01–0.55) and *P. mirabilis* (*p* = 0.02, OR = 9.16, 95% CI: 1.06–110.03). Nevertheless, the frequency of strong biofilm-producers was comparable between *P. aeruginosa* and *A. baumannii* strains.

Moderate biofilm-forming isolates (B++) showed similar frequencies among *K. pneumoniae*, *P. mirabilis* and *A. baumannii* isolates, whereas *P. aeruginosa* strains predominantly exhibited a strong biofilm-forming phenotype (B+++).

The study of the association between the resistance gene carriage and biofilm-forming ability was investigated separately for each species (Table 6). For the analysis, gene-carrying strains and biofilm-forming strains, were considered, respectively. A stratified analysis of biofilm formation intensity was not performed because over 80% of the strains presented a strong/moderate phenotype, so the analysis of levels would have low statistical power. However, it cannot be excluded that the relationship between gene portage and biofilm formation is dependent on its intensity.

Analysis of the association between resistance gene carriage and biofilm-forming capacity, as well as the strength of this relationship, demonstrated a strong, positive, and statistically significant association in *K. pneumoniae* strains. A similar result was obtained for *P. mirabilis* isolates.

With regard to *P. aeruginosa*, the Chi-square test indicated no significant association between the two variables, as all strains exhibited a biofilm-forming nature regardless of the presence or absence of antibiotic resistance genes.

A particular situation was also observed for *A. baumannii*, in which all isolates carried resistance genes. As a result, the biofilm-forming phenotype, which was variable among the strains, could not be interpreted in relation to this carriage.

Concerning *S. aureus*, the results show that these isolates were moderate biofilm-formers in over 57% of cases (Figure 3). Analysis of the association between resistance gene carriage and the biofilm-forming capacity did not reveal a significant relationship between these variables.

### 3.5. Biofilm Susceptibility to Antibiotics

Biofilm susceptibility to antibiotics was evaluated using the Calgary Biofilm Device. BMIC_50_ values were determined for all studied strains. The MIC for all planktonic forms of strains was also evaluated. The antibiotics tested were meropenem and amikacin.

The values obtained when testing biofilm and planktonic cells susceptibility for *K. pneumoniae*, *P. aeruginosa* and *A. baumannii* are presented in Table 7.

The differences between BMIC_50_ (for the biofilm form of strains) and MIC values (for planktonic form), obtained when testing the susceptibility to the two antibiotics, were analyzed using the Wilcoxon Signed-Rank test (Table 8). The test detects a general trend of BMIC_50_–MIC pairs (biofilm–planktonic cells) for each species studied, even if there are particular pairs with a different behavior compared to the general one observed through this test.

The BMIC_50_/MIC comparison cannot have clinical significance. In the case of biofilms, the BMIC value does not have standardized values for R/S. The test only indicates whether the biofilm is less susceptible to the antibiotics tested, without the statement being able to be clinically interpreted in relation to conventional S/R criteria used in antibiotic susceptibility testing.

Except for the *A. baumannii* testing to amikacin, the analyses showed that antibiotic susceptibility of biofilm forms of bacteria measured by BMIC_50_ were significantly lower than that of the planktonic bacteria measured by MIC. The Wilcoxon test indicated that the differences were statistically significant with a large effect size.

With regard to *A. baumannii*, testing to amikacin did not indicate a statistically significant difference between the MIC of planktonic strains and BMIC_50_. Nevertheless, the effect size was large (Table 8), suggesting a difference with potential biological relevance, but which did not reach the threshold of statistical significance due to the small sample size. The result is borderline, as the effect was large, but not statistically supported.

## 4. Discussion

Chronic non-healing foot ulcers are a major complication of causative diseases (diabetes mellitus, arterial ischemia, surgical wound, burn disease), contributing to the increase in morbidity and mortality in these pathologies [30]. The microbial population in chronic wounds exhibits great variability, determined by a combination of structural, physiological and environmental factors, which include genes carried, metabolic activity, intercellular interaction, host defense mechanisms and antibiotic exposure [31].

This study aimed to identify the carriage of a group of resistance genes and the presence of a phenotypic trait—biofilm formation—for the strains isolated from chronic non-healing wounds in hospital wards, to provide criteria for a more efficient therapeutic approach in the management of these chronic wounds. The selection of cases was made according to the specificity of the hospital infections.

In the study sample, the species with the highest frequency were the Gram-negative *K. pneumoniae* and *P. aeruginosa*, followed by *S. aureus*. These microorganisms have also been highlighted in other studies that reported the predominance of Gram-negative bacteria in chronic non-healing diabetic and non-diabetic wounds—*P. aeruginosa* as the main etiological factor, followed by *A. baumannii*, *Proteus* spp., *K. pneumoniae* and Gram-positive methicillin-resistant *S. aureus* [32,33,34].

Nevertheless, the dynamics of resistance phenotypes in these microorganisms are of particular importance and have been the subject of comparative analysis (2020vs2023) conducted by our group, focusing on the evolution of bacterial species isolated from clinical samples of burn patients [13]. The previous study demonstrated a marked increase in the multidrug-resistant (MDR) strains of *P. aeruginosa* from 18.52% in 2020 to 44.90% in 2023, with a similarly high prevalence observed in the present study (41.66%). An alarming rise in the pandrug-resistant (PDR) strains of *K. pneumoniae* was also noted, increasing from 5.55% to 40.91% (2020–2023); in our current study, XDR *K. pneumoniae* reached 70.37%, although no PDR strains were identified. With regard to *S. aureus*, the prevalence of methicillin-resistant strains (MRSA) increased from 41.18% in 2020 to 44.90% in 2023, reaching 52.63% in the current study. Similarly, MDR *S. aureus* strains showed an upward trend, from 58.82% in 2020 to 51.02% in 2023, and further to 68.42% in our research. Concerning the carbapenem-resistant phenotype (CR), an increasing trend was observed between 2020 and 2023, with further changes noted in the present study, as follows: *K. pneumoniae* (33.33–54.55% vs. 74.07%), *P. aeruginosa* (25.93–37.93% vs. 37.5%), and *A. baumannii* (91.30–88.24% vs. 85.71%) [13].

Detection of carbapenemases and β-lactamase genes by qualitative PCR can provide a molecular basis for the resistance phenotypes revealed by the antibiogram. However, this method only confirms the presence of resistance determinants, without providing information on their expression level. For some of the strains studied, the antibiogram was supplemented with rapid tests for the detection of cephalosporinases and carbapenemases production, which confirmed enzymatic activity. In these cases, it can be stated, at least to a certain extent, that there is a genotype–phenotype concordance at the functional level. Since these determinations were not performed for the entire batch of strains, the involvement of other resistance mechanisms cannot be excluded. Thus, the observed resistance phenotype can be determined both by the expression of the detected genes and by additional mechanisms, such as ESBL/AmpC production associated with the loss of porins and/or overexpression of efflux pumps [35,36].

The *aac(6′)-Ib* gene was identified in some strains in our study. Part of them presented the Ag resistance phenotype and others were sensitive to those antibiotics. The presence of the *aac(6′)-Ib* gene in susceptible strains can be explained by the mechanisms that condition the phenotypic expression of AG resistance, such as low expression levels, functional variability of the encoded enzyme, or lack of efficient enzymatic activity [37,38,39]. On the other hand, the absence of *aac(6′)-Ib* in aminoglycoside-resistant strains highlights the fact that the genetic determinism of AG resistance is complex and varied (AMEs genes, 16S rRNA methylase gene, MexXY efflux pump genes, etc.), requiring exhaustive research in this regard [38].

In our study, *K. pneumoniae* strains demonstrated a high carriage rate of resistance genes, oftentimes in multiple combinations, an aspect also reported in other studies. Thus, Smriti et al. reported the identification of *bla*NDM in 40% of *K. pneumoniae* isolates and the *bla*OXA 48 gene in 10% of strains, and the association of *bla*NDM + *bla*OXA48 together with an aminoglycoside-modifying enzyme (AME) was identified in 13.3% of cases [40]. Similarly, Latifi et al. showed that 83.9% of ESBL isolates expressed CTX-M type beta lactamase and 68.08% of those also harbored AME genes [41].

We identified a large group of XDR strains (approximately 70% isolates), for which the existence of multiple associations of carba- and non-carba *bla* genes was demonstrated. The carbapenemase-encoding genes carried by the strains were predominantly NDM and OXA, mostly of the OXA48 type. Isolates carrying the NDM-OXA and β-lactamase-encoding gene association accounted for approximately 50% of the *K. pneumoniae* strains. The presence of NDM-OXA48 is by far the most widespread type of carbapenemase association found in *K. pneumoniae* worldwide [42,43].

With regard to *P. aeruginosa* isolates, approximately 30% of the strains were carbapenem-resistant with resistance genes belonging to the OXA group (predominantly OXA23 and OXA24) without identification of NDM/VIM. Conversely, studies show that carbapenem resistance in *P. aeruginosa* is less often due to the presence of type D β-lactamases (OXA-type) and that it emerges more frequently as an expression of the carriage of metallo–β-lactamase genes—VIM/IMP/NDM [44,45]. In the OXA group, the most common are OXA-2-like and OXA-10-like, which we did not seek to identify [46,47]. Only one strain has exhibited the CR phenotype in the absence of a genetic determinant, which is explained by other mechanisms frequently found in this species, such as OprD loss, derepressed AmpC, and increased efflux [48,49]. The aminoglycoside resistance phenotype was isolated and detected in the absence of the sought-after *aac(6′)-Ib* gene. This behavior can be explained by the presence of another gene from the AME (Aminoglycoside-modifying enzyme) group, by low outer membrane permeability, active efflux, and rarely target modification [50]. On the other hand, we have identified a small number of strains carrying *aac(6′)-Ib*, all of which were sensitive to aminoglycosides. There are studies which show that the presence of the *aac(6′)-Ib* gene does not ensure phenotypic resistance to aminoglycosides. This is reliant on factors such as allelic variation and the level of gene expression, thus explaining the discrepancies between the genotypic presence and the MICs obtained phenotypically [39,51].

*A. baumannii* strains were highlighted by carrying an important set of genes, expressed by extensive antibiotic resistance, including resistance to carbapenems, found in most strains. The number of strains was small, but the predominance of group D β-lactamase genes (OXA-type) was observed, with the majority frequency of *bla*OXA23, as observed in other reports [44]. Studies show that *A. baumannii* preferentially carries NDM gene [52], which was also identified in our research, but with an isolated presence. Aminoglycoside resistance had high frequency, given the lack of identification of the *aac(6′)-Ib* resistance gene, indicating the existence of another genetic determinism.

Concerning the biofilm-forming capacity, this study has demonstrated that the isolates from chronic wounds are significant biofilm-formers. We have not identified strains that did not form biofilm, and 88.40% of the strains were strong/moderate biofilm-formers.

*P. aeruginosa* revealed a strong biofilm-forming capacity, which has also been noticed in other studies. Thi MT [53] has indicated that *P. aeruginosa* has a particular biofilm-forming potential compared to other bacteria, while Tuon FF [54] has suggested that biofilm formation is one of the most important determinants of pathogenicity in the case of *P. aeruginosa*. Biofilm protects the bacterium and contributes to the persistence of infections.

In our study, *K. pneumoniae*, *P. mirabilis* and *A. baumannii* proved to have at least a moderate biofilm-forming capacity in high proportions, similar in statistical significance.

In this regard, Seifi et al. [55] have noticed that *K. pneumoniae* shows different levels of biofilm production, highlighting its frequent ability to form biofilms. With regard to *P. mirabilis*, studies are related to urinary pathology and show the ability of the bacterium to form robust biofilms on artificial surfaces such as urinary catheters, due to its adhesion factors and motility [56,57]. Nonetheless, *P. mirabilis* is also detected in chronic wounds, in polymicrobial biofilm, along with *P. aeruginosa*, *S. aureus* and *Enterobacterales* species [6,58].

The results of this study demonstrated the existence of a strong and statistically significant association between resistance gene carriage and biofilm-forming capacity, applicable to *K. pneumoniae* and *P. mirabilis*.

This association can be explained, at least in part, by the linkage between antimicrobial resistance genes and biofilm-associated genetic determinants. Several studies have reported the co-existence of genes involved in biofilm formation and antimicrobial resistance. Silva-de-Jesus et al. demonstrated that, in MRSA strains, expression of the *ica*ADBC operon, which promotes the synthesis of a polysaccharide essential for biofilm development, is frequently associated with enhanced biofilm-forming capacity, thereby contributing to bacterial persistence and reduced susceptibility to antibiotic therapy [59]. Similarly, Salehi et al. identified quorum-sensing genes such as *lasI* and *lasR*, predominantly in clinical isolates of *P. aeruginosa* simultaneously resistant to multiple antibiotics. The authors also emphasized that the relationship between these genes and antimicrobial resistance requires further investigation [60]. There are also studies showing that antibiotic resistance genes are frequently located on the same mobile genetic elements (e.g., plasmids and integrons) as genetic determinants involved in biofilm formation, including those involved in adhesion (fimbriae, pili) and extracellular matrix production. In this regard, Norman et al. reported that the conjugative plasmid pOLA52 identified in *Escherichia coli* strains simultaneously carried genes encoding multidrug-resistance mediated by a resistance-nodulation-division (RND)-type efflux-pump (*oqxAB*) and genes involved in type 3 fimbriae formation (*mrkABCDF*), phenotypes typically associated with the chromosomes of opportunistic pathogens [61].

This type of co-location facilitates co-selection and, as a result, antimicrobial pressure not only selects resistance genes but may also indirectly maintain biofilm-associated genetic determinants. In addition, the biofilm matrix provides a highly favorable environment for horizontal gene transfer, thereby enhancing the dissemination of resistance determinants within bacterial communities [62]. The presence of extracellular DNA within the biofilm matrix together with high rates of plasmid-mediated gene transfer, further promotes this process [63]. As a result, the biofilm acts not only as a protective niche for microorganisms but also as a dynamic reservoir of resistance genes. These mechanisms may explain the persistence of infection and therapeutic failure observed in chronic wounds, even in cases where antibiogram-guided therapy is applied.

In the case of *P. aeruginosa* and *A. baumannii*, the test was not applicable for different and significant reasons. In the *P. aeruginosa* isolates, most strains (over 90%) showed a strong biofilm-forming capacity and under 10% of the strains displayed an average expression of this potential, indicating very low variability of this behavior. As a result, the presence of resistance genes proves not to be a condition for this potential. *P. aeruginosa* is a strong biofilm-forming bacterium and manifests this characteristic for both sensitive and antibiotic-resistant strains.

Concerning *A. baumannii* strains, they are mainly characterized by a high genetic carriage applicable to all strains and expressed by high antibiotic resistance behavior. As a result, this behavior is detected even if the strain is not biofilm-forming. Nevertheless, there are studies that show a significant correlation between multidrug resistance and biofilm formation for *A. baumannii* isolates [64].

*S. aureus* proved to be less equipped with the resistance genes, resistance behavior and phenotypes defined by the *mecA* gene. The biofilm-forming capacity of *S. aureus* was predominantly moderate without showing a relationship to the presence of genes at the strain level.

In our study, antimicrobial susceptibility to amikacin and meropenem was assessed for each isolate in both planktonic and biofilm states by determining MIC and BMIC_50_ values. Statistical analysis of the paired differences (BMIC_50_-MIC) demonstrated significantly reduced antibiotic susceptibility in the biofilm state. The only exception was *A. baumannii* tested to amikacin, where the differences were not statistically significant. This behavior has also been reported in previous studies [27,65] and can be explained by multiple factors influencing biofilm susceptibility including microbial species, biofilm age, density and thickness. Additional mechanisms are represented by physiological states of bacterial cells (inactive, persister, dormant), depletion of the antimicrobial agent in the surrounding fluid and limited penetration of the antimicrobial agents into the biofilm matrix, as described by Stewart [66].

The present study aimed to evaluate the carriage of resistance genes and the biofilm-forming capacity of strains identified in chronic wounds, as potential factors involved in treatment resistance, in order to suggest possible therapeutic strategies.

The study was not intended to provide a comprehensive characterization of antimicrobial resistance mechanisms. The molecular analysis was limited to a selected panel of resistance genes and therefore did not describe the full spectrum of resistance determinants. Consequently, the absence of the investigated genes should not be interpreted as the absence of resistance mechanisms.

The study indicated a high prevalence of resistance gene carriage among the isolated strains, as well as that many isolates exhibited strong/moderate biofilm-forming capacity. These two components contribute, to varying degrees, to treatment resistance in each non-healing wound, emphasizing the necessity for a customized therapeutic approach. The presence of wounds extensively colonized by biofilm proven by the strong biofilm-forming capacity of the isolated strains indicates the need for bolder local treatment strategies related to biofilm removal and disruption of its formation and protective mechanisms for the embedded bacterial population. Furthermore, knowledge of antibiotic resistance gene carriage provides additional information regarding the delayed healing of chronic wounds by highlighting certain mechanisms that are not revealed by conventional antimicrobial susceptibility testing results. These include the potential activation of resistance genes not expressed under standard laboratory testing conditions, horizontal gene transfer (predominantly plasmid-mediated) between bacteria, biofilm-facilitated resistance in the case of strains with a reduced load of resistance genes. Therefore, characterization of resistance genes may guide antibiotic therapy according to the potential for resistance adaptation and dissemination, as well as enable better correlation with the biofilm-colonized microenvironment of chronic wounds. This diagnostic approach can be achieved through a multiplex PCR-based investigation performed using rapid syndromic molecular diagnostic platforms which enable the rapid and simultaneous identification of pathogens and resistance genes directly from the clinical sample. Our laboratory has relevant experience in this field, including the use of the Unyvero platform (Curetis GmbH, Holzgerlingen, Germany); however, this type of testing has not yet been incorporated into the routine diagnostic workflow for these samples.

There are studies indicating that biofilms have a low susceptibility to antimicrobials through multiple mechanisms, both genetic and non-genetic. Genetic mechanisms include the facilitated horizontal gene transfer [67], quorum sensing systems [68], and regulatory pathway involving messengers such as cyclic di-AMP and cyclic di-GMP [69,70]. Non-genetic mechanisms include the presence of persistent cells and restricted penetration of antimicrobials in the biofilm [67]. These mechanisms contribute to antimicrobial tolerance and therapeutic failure.

Recent research on new therapeutic concepts shows the exceptional efficiency of aptamers in suppressing bacterial proliferation and stopping the evolution of biofilms in therapeutic environments [71]. Furthermore, Doherty et al. show that the association of the studied aptamer (αSA31 aptamer) with Vancomycin, reduced the distribution and persistence of MRSA in systemic infection, with an increase in the chances of survival [72]. Likewise, research is directed towards the study of bacterial resistance to protist grazing [73] and the mechanism of intervention of bacteriophages in biofilm [74] with the aim of using these biological agents in the treatment of chronic wound infections.

The present study presents several limitations as follows: it is a single-center study, conducted over a period of only 7 months, not all antibiotics of a class have been phenotypically tested nor have all genes that would induce antibiotics resistance, and the molecular analysis performed was limited to the qualitative detection of a selected panel of resistance genes. Also, no data linked to clinical outcomes were collected or analyzed. Despite these limitations, we believe that we have provided valuable information regarding the genotypic characterization of antimicrobial resistance, as well as the biofilm-forming capacity of isolates in chronic wounds, this being, to the best of our knowledge, the first study of this kind to be conducted in the western part of our country. Overall, the data obtained provide support for the integration of antibiofilm strategies into clinical protocols and for the development of targeted therapies, with the potential to improve clinical outcomes and limit the subsequent selection of resistant strains.

## 5. Conclusions

Gram-negative bacteria predominated among the chronic wounds isolated and analyzed, with most isolates exhibiting multidrug-resistant (MDR) or extensively drug-resistant (XDR) phenotypes. This pattern was particularly evident for *Klebsiella pneumoniae* and *Acinetobacter baumanii*, corroborating our previous results. The isolates were strong biofilm-formers, primarily *Pseudomonas aeruginosa* and *Acinetobacter baumanii.* A strong and statistically significant association was identified between resistance gene carriage and biofilm-forming capacity, particularly in *Klebsiella pneumoniae* and *Proteus mirabilis* isolates. In addition, significantly reduced antibiotic susceptibility was observed in the biofilm state. The association and potential co-transfer of antimicrobial resistance genes with biofilm-forming genetic determinants may contribute to enhanced treatment resistance and persistence of chromic wound infection. These findings highlight the need for personalized therapeutic strategies in chronic wound management, considering the mechanisms underlying treatment resistance. These include bacterial factors and the wound microenvironment colonized by the biofilm and the relative contribution of each to the overall resistance profile.

## Figures and Tables

**Table 1:** PCR primer sets used for Gram-negative and Gram-positive strains.

Gram-Negative Primer Sets		Size (bp)	Ref.
*blaTEM*	F: TTGCACAACATGGGGGATC, R: AGCTAGAGTAAGTAGTTCGCCAGTTAATAGTT Probe FAM-AACCGGAGCTGAATGAAGCCATACCAA BHQ_1	108	[16]
*blaSHV*	F: CGATAACAGCGCCGCC, R: TTCCCAGCGGTCAAGGC Probe: FAM-TGACTGCCTTTTTGCGCCAGATCG BHQ_1	109	[16]
*blaCTX-M-1gr*	F-CTGGGTGTGGCATTGATTAACA, R-CTCGCTGATTTAACAGATTCGGTT Probe MGB-ATGGCCGTGGCCG-FAM	151	[16]
*blaOXA-48*	F: TGTTTATCAAGAATTTGCCCGC, R: TTCGGTCAGCATGGCTTGT Probe MGB-CGACGGTGGTATTCGA-FAM	232	[16]
*blaOXA-23*	F GACACTAGGAGAAGCCATGAAG; R CAGCATTACCGAAACCAATAC probe FAM-CCAGTCTATCAGGAACTTGCGCGA-BHQ_1	116	[17]
*blaOXA-24*	F GATGACCTTGCACATAACCG; R CAGTCAACCAACCTACCTGTG Probe FAM-AGTAACACCCATTCCCCATCCACTTTT- IABkFQ	151	[17]
*blaNDM*	F: ATTAGCCGCTGCATTGAT, R: CATGTCGAGATAGGAAGTG Probe 5-FAM-CTG [+C]CA [+G]AC [+A]TT [+C]GG TGC-BHQ_1 LNA	154	[18]
*blaVIM*	F: GAGTTGCTTTTGATTGATACAG, R: TCGATGAGAGTCCTTCTAGA	247	[17]
*aac(6′)-Ib*	F: AACTTGCGAGCGATCCGA, R: TGGCGTGTTTGAACCATGTAC Probe QSY-TACCTTGCTTCTCAAACCCCGCTTTCTC-JUN	101	[16]
Gram-positive primer sets			
*mecA*	F CAATGCCAAAATCTCAGGTAAAGTG, R AACCATCGTTACGGATTGCTTC Probe FAM-ATGAGCTATATGAGAACGG-MGBNFQ	147	[ST]
*blaZ*	F: GCTTTAAAAGAACTTATTGAGGCTTCA; R: CCACCGATYTCKTTTATAATTT probe, FAM AGTGATAATACAGCAAACAA MGBNFQ	233	[19]
*gyrA83*	F TACCATCCCCATGGTGACTC, R: GCCATGCGGACAATCGTGTC	440	[20]
*ermA*	F: AAGCGGTAAACCCCTCTGA, R: TTCGCCATTTGGGGAGACT	421	[21]
*ermB*	F CATTTAACGACGAAACTGGC, R: GGAACATCTGTGGTATGGCG	425	[22]
*aac(6′)/aph(2″)*	F TACAGAGCCTTGGGAAGATG, R: CATTTGTGGCATTATCATCATATC	406	[23]

Legend: F = Forward primer; R = Reverse primer. Forward and reverse primer sequences are shown in the 5′–3′ direction; ST: This study—Primers targeting the *mecA* gene were obtained from integrated DNA Technologies (IDT, Leuven, Belgium).

**Table 2:** Distribution of bacterial species within the study sample (*n* = 88).

Species	*K. pneumoniae*	*P. aeruginosa*	*P. mirabilis*	*A. baumannii*	*S. aureus*
% (*n*)	39.13 (27)	34.78 (24)	15.94 (11)	10.14 (7)	21.60 (19)

**Table 3:** Antimicrobial resistance genes identified in Gram-negative isolates.

Species	*bla* TEM	*bla* SHV	*bla* CTX-M	β-Lactamase	*bla* OXA-23	*bla* OXA-24	*bla* OXA-48	*bla* NDM	*bla* VIM	Carbapenemase	*aac(6′)-Ib*	With Genes
K.P 27 % (no.)	77.77 (21)	85.18 (23)	74.07 (20)	88.88 (24)	14.81 (4)	18.51 (5)	44.44 (12)	70.37 (19)	0.00 (0)	77.77 (21)	0.00 (0)	92.59 (25)
P.A 24 % (no.)	25 (6)	12.5 (3)	4.16 (1)	33.33 (8)	16.66 (4)	20.83 (5)	4.16 (1)	0.00 (0)	0.00 (0)	33.33 (8)	8.33 (2)	50 (12)
P.M 11 % (no.)	72.72 (8)	0.00 (0)	18.18 (2)	72.72 (8)	9.09 (1)	0.00 (0)	18.18 (2)	9.09 (1)	0.00 (0)	27.27 (3)	18.18 (2)	72.72 (8)
A.B 7 % (no.)	85.71 (6)	14.28 (1)	14.28 (1)	100 (7)	71.42 (5)	14.28 (1)	14.28 (1)	28.57 (2)	0.00 (0)	85.71 (6)	14.28 (1)	100 (7)

Legend: K.P: K. pneumoniae, P.A: P. aeruginosa, P.M: P. mirabilis, A.B: A. Baumannii.

**Table 4:** Antimicrobial resistance genes identified in *S. aureus* isolates (*n* = 19).

*S. aureus*	*bla*Z	*mecA*	*Gyr A83*	*aac(6′)aph(2″)*	*ermA*	*ermB*	With Genes
% (*n*)	5.26 (1)	52.63 (10)	5.26 (1)	15.78 (3)	0.00 (0)	5.26 (1)	57.89 (11)

**Table 5:** Antimicrobial resistance phenotypes identified among Gram-negative strains.

Species	AG-R	ESBL	CR	MDR	XDR
K.P 27, % (nr.)	51.85 (14)	77.77 (21)	74.07 (20)	85.18 (23)	70.37 (19)
P.A 24, % (nr.)	12.5 (3)	/	37.5 (9)	41.66 (10)	8.33 (2)
P.M 11, % (nr.)	27.27 (3)	0.00 (0)	0.00 (0)	63.63 (7)	0.00 (0)
A.B 7, % (nr.)	71.42 (5)	/	85.71 (6)	100 (7)	85.71 (6)

Legend: K.P: *K. pneumoniae*, P.A: *P. aeruginosa*, P.M: *P. mirabilis*, A.B: *A. baumannii*, AG-R: aminoglycoside resistance, ESBL: extended-spectrum β-lactamase, CR—carbapenem resistance, MDR: multidrug resistance, XDR: extensively drug-resistant, (/): not applicable.

**Table 6:** Analysis of the association between the resistance gene carriage and the biofilm-forming capacity in Gram-negative and Gram-positive strains.

Study Association	Fisher’s Exact Test (*p*)	Cramer’s V	Interpretation
*K. pneumoniae* with resistance genes/biofilm-forming capacity	*p* = 0.009	V = 0.593	Strong and positive, statistically significant
*P. aeruginosa* with resistance genes/biofilm-forming capacity	*p* = 1	V = 0.00	There is no relationship between the variables
*P. mirabilis* with resistance genes/biofilm-forming capacity	*p* = 0.022	V = 0.833	Strong and positive, statistically significant
*A. baumannii* with resistance genes/biofilm-forming capacity	No statistics are computed because one variable is constant.		/
*S. aureus* with resistance genes/biofilm-forming capacity	*p* = 0.377	V = 0.320	Statistically insignificant association

**Table 7:** BMIC_50_ and MIC values (≥) obtained for antibiotics tested against planktonic and biofilm forms of the isolates. The meaning of the values is greater than or equal (≥).

*K. pneumoniae*				*P. aeruginosa*				*A. baumanii*			
MEM BMIC_50_	MEM MIC	AK BMIC_50_	AK MIC	MEM BMIC_50_	MEM MIC	AK BMIC_50_	AK MIC	MEM BMIC_50_	MEM MIC	AK BMIC_50_	AK MIC
128	16	128	32	4	4	2	64	32	16	32	32
1	0.25	64	2	32	16	64	64	64	16	128	64
128	16	64	4	16	0.25	64	4	16	8	64	8
16	16	64	32	0.5	1	8	2	16	16	64	64
16	16	8	32	4	0.25	2	2	32	16	128	64
16	8	8	8	0.25	0.25	4	4	128	32	128	64
16	8	128	16	0.5	0.5	8	4	32	8	32	32
8	16	128	32	0.12	0.25	0.25	4	/	/	/	/
32	8	128	16	32	16	64	64	/	/	/	/
64	16	64	32	2	1	32	2	/	/	/	/
0.5	8	1	16	8	8	32	16	/	/	/	/
128	16	128	32	0.5	0.5	2	2	/	/	/	/
16	16	128	32	2	0.25	2	2	/	/	/	/
0.25	0.25	2	2	2	1	2	2	/	/	/	/
16	16	64	32	2	0.5	16	2	/	/	/	/
32	16	1	32	2	1	32	2	/	/	/	/
128	16	128	32	2	1	32	8	/	/	/	/
32	16	64	32	32	1	8	2	/	/	/	/
32	0.25	4	4	8	4	32	16	/	/	/	/
16	16	64	32	1	1	4	2	/	/	/	/
8	16	64	32	8	0.5	8	2	/	/	/	/
128	16	32	32	4	4	8	4	/	/	/	/
0.5	0.25	64	2	16	0.25	128	4	/	/	/	/
0.12	0.25	1	2	2	1	32	4	/	/	/	/
0.25	0.25	2	1	/	/	/	/	/	/	/	/
32	16	128	32	/	/	/	/	/	/	/	/
16	16	1	32	/	/	/	/	/	/	/	/

Legend: MEM: meropenem; AK: amikacin, BMIC_50_: minimum antibiotic concentration that inhibits 50% of the biofilm growth, MIC: minimum inhibitory concentration. (/): indicates the absence of a tested strain.

**Table 8:** Difference in meropenem and amikacin susceptibility between planktonic and biofilm states of Gram-negative strains.

Study Association	z	*p*	r	Interpretation
*K. pneumoniae* MEM BMIC_50_/MIC	3.109	0.002	0.598	Significant, large effect size
*K. pneumoniae* AK BMIC_50_/MIC	3.620	<0.001	0.696	Significant, large effect size
*P. aeruginosa* MEM BMIC_50_/MIC	3.490	<0.001	0.712	Significant, large effect size
*P. aeruginosa* AK BMIC_50_/MIC	2.772	0.006	0.565	Significant, large effect size
*P. mirabilis* MEM BMIC_50_/MIC	2.911	0.004	0.877	Significant, large effect size
*P. mirabilis* AK BMIC_50_/MIC	2.032	0.042	0.612	Significant, large effect size
*A. baumannii* MEM BMIC_50_/MIC	2.060	0.039	0.778	Significant, large effect size
*A. baumannii* AK BMIC_50_/MIC	1.890	0.059	0.714	Statistically insignificant

Legend: MEM: meropenem; AK: amikacin, BMIC_50_: minimum antibiotic concentration that inhibits 50% of the biofilm-growth, MIC: minimum inhibitory concentration, r = effect size: ≥0.5 large effect.

## Data Availability

The data supporting the findings of this study are available from the corresponding author upon reasonable request.

## Abbreviations

MIC	Minimum inhibitory concentration
Real-time PCR	Real-time Polymerase Chain Reaction
MEM	Meropenem
AK	Amikacin
GPC	Gram-positive Cocci
GNB	Gram-negative bacilli
GP	Gram-positive
GN	Gram-negative
MRSA	Methicillin-resistant *Staphylococcus aureus*
MR	Methicillin-resistant
XDR	Extensively drug-resistant
MDR	Multidrug-resistant
ESBL	Extended-spectrum β-Lactamase
OD	Optical density
QC	Quality control
ODc	Cut-off optical density
